# Assessment of Preoperative Serum Vitamin D Levels in Differentiated Thyroid Cancer

**DOI:** 10.7759/cureus.116490

**Published:** 2026-09-18

**Authors:** Abdulaziz A Alsalem, Abdulaziz A Alnasser, Thamer A Althunayan, Norah H Aldawsari, Abdulaziz Alaraifi, Mohammed Alessa

**Affiliations:** 1 Division of Otolaryngology - Head and Neck Surgery, Department of Surgery, King Abdulaziz Medical City, Ministry of National Guard Health Affairs, Riyadh, SAU; 2 Department of Research, King Abdullah International Medical Research Center, Riyadh, SAU; 3 College of Medicine, King Saud bin Abdulaziz University for Health Sciences, Riyadh, SAU; 4 Department of Otolaryngology - Head and Neck Surgery, College of Medicine, King Saud University, Riyadh, SAU

**Keywords:** 25-hydroxyvitamin d, differentiated thyroid cancer, thyroid nodules, vitamin d deficiency, vitamin d supplementation

## Abstract

Introduction: With the rising incidence of differentiated thyroid cancer (DTC), identifying its risk factors has become increasingly important. Vitamin D deficiency (VDD) is a recognized risk factor for several other types of cancer. Objective: This study aims to assess the association between preoperative serum 25-hydroxyvitamin D (25(OH)D) levels and DTC and, secondarily, to evaluate the association between vitamin D (VIT D) supplementation and DTC. Materials and methods: This retrospective case-control study included patients who underwent thyroidectomy at a tertiary care institute from January 2016 to January 2022. Preoperative serum 25(OH)D levels and histopathology of the resected thyroid were evaluated. Multivariable logistic regression analysis was performed to determine the association between serum 25(OH)D levels and DTC. Results: A total of 633 patients were included (mean age, 46.83 ± 13.8 years; 530 (83.7%) were female), comprising 325 patients with malignant thyroid nodules and 308 patients with benign thyroid nodules who served as controls. The malignant group had significantly higher serum 25(OH)D levels than the controls (p = 0.037). However, 25(OH)D categories (deficient, inadequate, normal, and high) did not differ significantly between groups. Patients taking VIT D supplements were less likely to have DTC than those not taking supplements (45.5% vs. 57.5%, p = 0.003). In multivariable regression analysis, patients who took VIT D supplements had 37% lower odds of DTC (OR = 0.634; 95% CI = 0.460-0.873). Conclusions: Serum 25(OH)D levels were not significantly associated with DTC. However, VIT D supplementation was independently associated with lower odds of DTC.

## Introduction

Thyroid cancer is the most prevalent endocrine malignancy, accounting for 3-4% of all cancers [1]. The incidence of thyroid cancer in Saudi Arabia (SA) increased 26-fold between 1990 and 2016 [2]. Differentiated thyroid cancer (DTC) is the most common form of thyroid cancer, accounting for approximately 90% of all thyroid carcinoma cases [3]. The etiology and pathophysiology of DTC are still being investigated, with some risk factors identified and others still under investigation. The association between vitamin D (VIT D) status and DTC has not yet been thoroughly investigated. Although a clear association exists between VIT D deficiency (VDD) and multiple cancers, its effect on DTC remains ambiguous and controversial.

The effect of VIT D on calcium and phosphate homeostasis is well known; however, little is known about its effects on proliferation, differentiation, and cell-cycle regulation [4]. Calcitriol is the active form of VIT D. It is considered a steroid molecule because it can freely diffuse through the lipid bilayer of the cell membrane and bind to its receptor in the nucleus, initiating cascades that regulate specific gene expression and the cell cycle [4]. VDD has been reported as a possible risk factor for breast, prostate, and colon cancers [5].

The relationship between VIT D and DTC has been investigated in both in vitro and in vivo studies. Clinical studies have yielded conflicting results, although several studies have reported an inverse association between VIT D levels and DTC [6,7]. However, other studies found no association between VIT D levels and DTC [8]. Epidemiological studies evaluating VIT D supplementation and other lifestyle-related exposures have also reported inconsistent findings [9-11]. For example, a survey by Greenlee et al. relied on patient-reported VIT D supplementation or documented VIT D prescriptions rather than direct measurement of serum 25(OH)D levels [9].

No studies have examined the relationship between VIT D levels and DTC in SA patients. Given the high prevalence of VDD in SA, the lack of local evidence, and inconsistent international findings, further investigation into the association between VIT D levels and DTC is warranted. This study primarily aimed to assess the association between preoperative serum 25-hydroxyvitamin D (25(OH)D) levels and DTC. The secondary objective was to evaluate the association between VIT D supplementation and DTC.

This article was previously posted to the Research Square preprint server on January 31, 2023.

## Materials and methods

A retrospective case-control study was conducted among patients aged 18 years or older who underwent hemithyroidectomy or total thyroidectomy at a tertiary care institute in Riyadh, SA, between January 2016 and January 2022. The study was reported in accordance with the Strengthening the Reporting of Observational Studies in Epidemiology (STROBE) guidelines [12]. Indications for thyroidectomy and patient management were based on the 2015 American Thyroid Association (ATA) guidelines [13]. Thyroidectomies were performed by multiple surgeons at the study institution. Patients without preoperative serum 25(OH)D measurements were excluded. The King Abdullah International Medical Research Center approved the study on September 29, 2021 (approval number: H-01-R-005). The study was exempt from the informed consent requirement because of its retrospective nature.

Patients' electronic medical records were reviewed using the hospital's electronic health record system. Data collected included demographic characteristics, comorbidities (e.g., history of other malignancies, diabetes mellitus, renal disease, and hyperparathyroidism), and laboratory parameters, including preoperative serum 25(OH)D, parathyroid hormone, and adjusted calcium levels. Histopathological examinations of the surgical specimens were performed by multiple histopathologists as part of routine clinical practice. The final histopathological diagnosis was obtained from the pathology reports in the electronic medical records and was used to classify thyroid nodules as benign or malignant.

We analyzed the data using SPSS Statistics version 25.0 (IBM Corp. Released 2017. IBM SPSS Statistics for Windows, Version 25.0. Armonk, NY). Categorical variables were summarized as frequencies and percentages, and continuous variables as means and standard deviations (SD). Patients were categorized into benign and malignant groups according to the final histopathological diagnosis. Categorical and continuous variables were compared using the chi-square test and the independent-samples t-test, respectively. Variables of interest were entered into a multivariable logistic regression model to identify independent factors associated with thyroid malignancy. A two-sided p < 0.05 was considered statistically significant.

## Results

The study included 633 patients: 308 with benign thyroid nodules and 325 with malignant thyroid nodules. The mean age was 46.83 ± 13.8 years, and most patients were female (n = 530, 83.7%). The mean serum 25(OH)D level was 48.71 ± 29.9 nmol/L, and 51 patients (8.1%) had VDD. Table 1 presents the participants' clinical characteristics.

**Table 1:** Clinical characteristics of the study group BMI: body mass index, DM: diabetes mellitus, CKD: chronic kidney disease, ESRD: end-stage renal disease, 25(OH)D: 25-hydroxyvitamin D, PTH: parathyroid hormone, VIT D: vitamin D, SD: standard deviation

Variables	Overall (n = 633)
Age (mean ± SD)	46.83 (±13.8)
Gender (N%)	
Male	103 (16.3%)
Female	530 (83.7%)
BMI (mean ± SD)	31.67 (±6.7)
History of cancer (N%)	34 (5.4%)
DM (N%)	194 (30.6%)
CKD/ESRD (N%)	15 (2.4%)
Hyperparathyroidism (N%)	
Primary	25 (3.9%)
Secondary	120 (19.0%)
Serum 25(OH)D (mean ± SD)	48.71 nmol/L (±29.9)
Serum 25(OH)D (N%)	
Deficient	51 (8.1%)
Inadequate	112 (17.7%)
Normal	419 (66.2%)
High	51 (8.1%)
VIT D supplements (N%)	
Yes	325 (51.3%)
No	308 (48.7%)
PTH (mean ± SD)	10.47 pmol/L (±14.7)
Adjusted calcium (mean ± SD)	2.31 mmol/L (±0.2)

Table 2 summarizes the differences between the benign and malignant thyroid groups. The malignant group had a significantly higher mean age and serum 25(OH)D level than the benign group (48.31 vs. 45.26 years; p = 0.006; 51.12 vs. 46.17 nmol/L; p = 0.037). However, when serum 25(OH)D levels were categorized as deficient, inadequate, normal, or high, the two groups showed no significant differences. A significantly lower proportion of patients in the malignant group were taking VIT D supplements than in the benign group (148/325 (45.5%) vs. 177/308 (57.5%)). Additionally, the prevalence of diabetes mellitus and a history of malignancy were significantly higher in the malignant group.

**Table 2:** Description of selected characteristics of benign and malignant thyroid nodules * Significant at p < 0.05 level. BMI: body mass index, DM: diabetes mellitus, CKD: chronic kidney disease, ESRD: end-stage renal disease, 25(OH)D: 25-hydroxyvitamin D, PTH: parathyroid hormone, SD: standard deviation

Variables	Benign (n = 308)	Malignant (n = 325)	p-value
Age (mean ± SD)	45.26 (±13.29)	48.31 (±14.16)	0.006*
Gender (N%)			
Male	50 (16.2%)	53 (16.3%)	0.980
Female	258 (83.8%)	272 (83.7%)	
BMI (mean ± SD)	31.70 (±6.71)	31.65 (±6.75)	0.923
History of cancer (N%)	7 (2.3%)	27 (8.3%)	0.001*
DM (N%)	81 (26.3%)	113 (34.8%)	0.021*
CKD/ESRD (N%)	5 (1.6%)	10 (3.1%)	0.229
Hyperparathyroidism (N%)			
Primary	12 (3.9%)	13 (4.0%)	0.957
Secondary	57 (18.5%)	63 (19.4%)	
Serum 25(OH)D (mean ± SD)	46.17 nmol/L (±24.19)	51.12 nmol/L (±34.39)	0.037*
Serum 25(OH)D nmol/L (N%)			
Deficient	29 nmol/L (9.4%)	22 nmol/L (6.8%)	0.236
Inadequate	59 nmol/L (19.2%)	53 nmol/L (16.3%)	
Normal	192 nmol/L (62.3%)	227 (69.8%)	
High	28 nmol/L (9.1%)	23 (7.1%)	
Vitamin D supplements (N%)	177 (57.5%)	148 (45.5%)	0.003*
PTH (mean ± SD)	9.78 pmol/L (±10.42)	11.12 pmol/L (±17.87)	0.413
Adjusted calcium (mean ± SD)	2.32 mmol/L (±0.17)	2.30 mmol/L (±0.14)	0.316

Variables that were significant in the univariate analysis were entered into the multivariable logistic regression model (Table 3). Patients taking vitamin D supplements had 37% lower odds of DTC than those not taking supplements (OR = 0.634; 95% CI = 0.460-0.873; p = 0.005). Patients with a history of malignancy had 3.28-fold higher odds of malignant thyroid nodules (OR = 3.276; 95% CI = 1.385-7.746; p = 0.007). Age, serum 25(OH)D level, and diabetes mellitus were not independently associated with DTC in the multivariable logistic regression analysis.

**Table 3:** Predictors of thyroid cancer in logistic regression analysis * Significant at p < 0.05 level. DM: diabetes mellitus, 25(OH)D: 25-hydroxyvitamin D, OR: odds ratio, LCI: lower confidence interval, UCI: upper confidence interval, VIT D: vitamin D

Variable	OR	p-value	95% LCI	95% UCI
Age	1.010	0.154	0.996	1.023
Serum 25(OH)D	1.003	0.267	0.998	1.009
History of cancer	3.276	0.007*	1.385	7.746
DM	1.239	0.272	0.846	1.814
VIT D supplements	0.634	0.005*	0.460	0.873

## Discussion

Thyroid cancer is associated with substantial medical and psychological morbidity. Identifying a modifiable risk factor or biomarker is important for preventing disease and enabling early diagnosis. In the present study, preoperative VIT D supplementation was significantly associated with lower odds of DTC. In a case-control study by Mack et al., women aged 15-54 years who took weekly VIT D supplements showed no change in DTC risk [11]. In contrast, an interview study reported a higher risk of DTC in both men and women who regularly consumed VIT D supplements [10]. The Vitamins and Lifestyle (VITAL) Study cohort reported that VIT D supplementation was associated with DTC [9]. However, Zhang et al. and the National Institutes of Health-American Association of Retired Persons reported that the evidence regarding the association between dietary VIT D intake or supplementation and DTC remains inconclusive [14]. Despite the limited data from human observational studies on the association between VIT D supplementation and DTC, several in vitro and animal studies support our findings [13-15]. This inverse association may be explained by the antiproliferative effects of VIT D and its ability to promote apoptosis in both differentiated and undifferentiated thyroid cancer cells [15-17]. Additionally, animal studies have shown that VIT D supplementation is associated with a significant reduction in tumor burden by modulating gene expression and signaling pathways [17].

Our study found no significant association between serum 25(OH)D levels and DTC. This finding is consistent with several studies reporting that serum 25(OH)D levels were not associated with the risk of malignant thyroid disease or adverse clinicopathological features [18-20]. Jonklaas et al. demonstrated that preoperative 25(OH)D levels were not associated with the diagnosis, tumor stage, tumor size, or extrathyroidal extension of DTC [18]. Furthermore, Laney et al. reported similar rates of VDD among patients with benign thyroid nodules, DTC, and DTC in remission [20]. Another study found no association between papillary thyroid carcinoma and VDD when compared with patients with Hashimoto’s thyroiditis [19]. Conversely, several studies have reported that low circulating 25(OH)D levels are associated with DTC [7,21,22]. A meta-analysis of 14 studies found significantly lower preoperative serum 25(OH)D levels in patients with DTC than in controls [23]. Similarly, Stepien et al. reported significantly lower serum 25(OH)D concentrations in patients with papillary, follicular, and anaplastic thyroid carcinoma, with VIT D levels varying according to tumor stage [24]. Other studies have also linked low VIT D levels with more advanced thyroid malignancy [7,23,25]. In contrast, one study reported higher serum 25(OH)D levels among patients with papillary thyroid carcinoma [26]. Variability among these findings may reflect differences in VDD definitions, units of measurement, laboratory assays, and study methodologies [25].

The timing of preoperative serum 25(OH)D measurement may also have contributed to the inconsistent findings across studies. In our study, we used the most recent preoperative serum 25(OH)D level, regardless of the interval before surgery. Hu et al. measured serum 25(OH)D immediately before surgery, whereas Sulibhavi et al. used the lowest recorded serum 25(OH)D level during the year preceding thyroidectomy [6,22]. Neither these studies nor ours evaluated the chronicity of VDD. Long-term VIT D status may be more informative than a single serum 25(OH)D measurement when investigating its relationship with thyroid malignancy.

Patients with a history of malignancy had significantly higher odds of malignant thyroid nodules in our cohort. A Taiwanese study estimated the incidence of second primary malignancies among patients with DTC to be approximately 9.13% [27]. Similarly, a Saudi Arabian study reported a prevalence of approximately 5.0% for second primary malignancies among patients with DTC [28].

This study has several limitations. First, its retrospective single-center design may limit the generalizability of the findings and preclude establishing a causal relationship between VIT D and DTC. Second, missing or undocumented data, including the duration and dose of VIT D supplementation and the chronicity of VDD, may have affected the accuracy of our findings. Despite these limitations, this study evaluated several potential risk factors for malignant thyroid nodules that have not previously been investigated in the Saudi population, including serum 25(OH)D levels and VIT D supplementation.

## Conclusions

Investigating potentially modifiable factors associated with DTC is important given its increasing incidence. In this study, VIT D supplementation was significantly associated with lower odds of DTC, whereas a history of another primary malignancy was associated with higher odds of malignant thyroid nodules. Serum 25(OH)D levels were not independently associated with DTC. These results add to the limited evidence regarding the relationship between VIT D and thyroid malignancy in the Saudi population. However, because this was a retrospective case-control study, causality cannot be established. Further prospective multicenter studies with longitudinal assessment of VIT D status and supplementation are needed to further clarify these associations.
